# Physicochemical Characterization of a Biomimetic, Elastin-Inspired Polypeptide with Enhanced Thermoresponsive Properties and Improved Cell Adhesion

**DOI:** 10.1021/acs.biomac.3c00782

**Published:** 2023-10-27

**Authors:** Antonella Bandiera, Laura Colomina - Alfaro, Paola Sist, Giovanna Gomez d’Ayala, Federica Zuppardi, Pierfrancesco Cerruti, Ovidio Catanzano, Sabina Passamonti, Ranieri Urbani

**Affiliations:** † Department of Life Sciences, University of Trieste, via L. Giorgieri, 1, 34127 Trieste, Italy; ‡ Institute for Polymers, Composites and Biomaterials (IPCB-CNR), Via Campi Flegrei 34, 80078 Pozzuoli, NA, Italy; § Department of Chemical and Pharmaceutical Sciences, University of Trieste, via L. Giorgieri, 1, 34127 Trieste, Italy

## Abstract

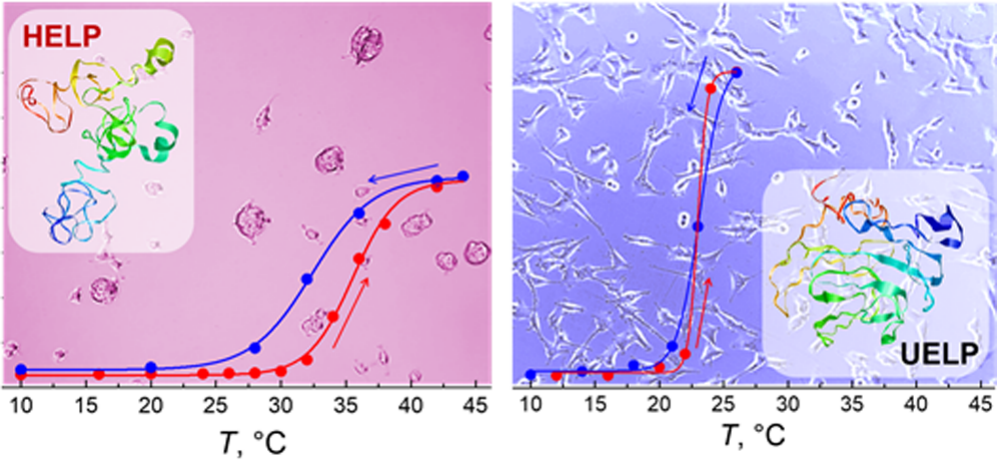

Genetic engineering allows fine-tuning and controlling protein properties, thus exploiting the new derivatives to obtain novel materials and systems with improved capacity to actively interact with biological systems. The elastin-like polypeptides are tunable recombinant biopolymers that have proven to be ideal candidates for realizing bioactive interfaces that can interact with biological systems. They are characterized by a thermoresponsive behavior that is strictly related to their peculiar amino acid sequence. We describe here the rational design of a new biopolymer inspired by elastin and the comparison of its physicochemical properties with those of another already characterized member of the same protein class. To assess the cytocompatibility, the behavior of cells of different origins toward these components was evaluated. Our study shows that the biomimetic strategy adopted to design new elastin-based recombinant polypeptides represents a versatile and valuable tool for the development of protein-based materials with improved properties and advanced functionality.

## 1 Introduction

Elastin is one of the main structural components of tissues that undergoes countless cycles of expansion and contraction during the lifetime of vertebrates. For this reason, it represents a valuable model to get inspiration for the design and realization of biomaterials with advanced functionality and properties.^1^

Elastin-like polypeptides (ELPs) are recombinant proteins modeled after elastin, mimicking its repetitive structure. Resembling the bovine elastin exon 18 sequence, the ELPs are constituted by long stretches of the regularly repeated VPGVG pentapeptidic motif, which is responsible for the outstanding inverse phase transition behavior that characterizes elastin and these polypeptides.^2^

In the past decade, our group focused on the human elastin homologue that shows a regularly repeated stretch of hexapeptidic rather than pentapeptidic motifs, these last being less represented and interspersed throughout its primary structure. With the aim to realize something between a protein and a polymer, following a biomimetic approach, we adopted the exon 23 and 24 amino acid sequences as the basic monomer to be reiterated. The former corresponds to a cross-linking domain, and the latter consists of the repeated hexapeptidic VAPGVG stretch, resulting in the human elastin-like polypeptide (HELP) family.^3^ These versatile biopolymers were described and characterized, and a method to obtain a hydrogel matrix was set up.^4^ HELP was also further modified by clonal fusion with different bioactive domains, representing a valuable carrier to increase the yield of difficult-to-express or active peptides.^5^ The HELP and its modifications showed no pro-inflammatory activity and good cytocompatibility, especially toward myoblast cells.^5a,6^ However, cell-type-dependent adhesion on HELP-based substrates was observed.^6,7^ Although the HELP-derived hydrogel matrices showed no cytotoxicity, the cell adhesion on the HELP-based scaffold was improved by the addition of pro-adhesive sequences.^6,8^ Moreover, some issues may arise because the HELP elastin-like sequence characterizing the human homologue may elicit an immune response in other organisms, like animal models being used to evaluate the compatibility of biomaterials where this sequence is not present.^9^ For example, antibodies that recognize the VAPGVG motif were successfully raised in mice.^10^ Last, the chemotactic activity of this same motif is well-known,^11^ and this should be considered for the development of new biomaterials intended for prolonged contact with tissues and organs. The perspective to broaden the compatibility toward as many cell types as possible and, more generally, toward different organisms still maintaining immunotolerance and the potential as carrier fusion partners delineated our approach. Thus, to further extend the properties of the biopolymer and, hence, those of the derived materials, we undertook the assembly of a new ELP biopolymer.

In this paper, we describe the design of the sequence and the production of this construct, as well as its physicochemical characterization. The behavior of this biopolymer was compared with that of the previously described HELP prototype by analyzing it with different techniques, such as turbidimetric analysis, circular dichroism, dynamic light scattering, and nuclear magnetic resonance. The response of cells to surfaces conditioned with these recombinant biopolymers was also evaluated.

## 2 Material and Methods

### 2.1 UELP Biopolymer Cloning and Production

The “universal” ELP (UELP) coding sequence was assembled following the same strategy already adopted for the HELP synthetic gene.^12^ Briefly, the nucleotidic sequence of 413 bp coding for a tandem repeat of the HELP cross-linking domain and the sequence coding for the nonapeptidic repeats inspired by the human exon 26 were the basic modules constituting the monomer to be reiterated. This sequence, flanked by the *Bam*HI and *BglI* restriction sites at the 5′ and by *DraIII* and *Hin*dIII at the 3′ end, was designed, optimized for *Escherichia coli* expression, and synthesized (Eurofins Genomics). Both the synthetic sequence and the pEX8EL plasmid for HELP expression were digested with *Bam*HI*/HindIII* to replace the HELP gene with the first UELP monomer. This latter was doubled by in-frame inserting another monomer by recombination of *BglI/DraIII* ends, cutting the vector with *DraIII*. After one more round of duplication, exploiting the same restriction sites, the UELP gene coding for eight cross-linking domains alternating with 8 hydrophobic domains was obtained and verified by sequencing (Eurofins Genomics).

Expression in *E. coli* C3730 and purification of the recombinant UELP and HELP biopolymers were carried out under standard conditions as previously described.^13^

### 2.2 Physicochemical Characterization

#### 2.2.1 Secondary Structure Evaluation

Using the ProtParam (Expasy) program available on the SIB Swiss Institute of Bioinformatics (https://www.expasy.org/) the grand average of hydropathy value (GRAVY) for proteins was calculated. This parameter was obtained as the sum of hydropathy values of all of the amino acids divided by the number of residues in the sequence.

Prediction of secondary structures of UELP was based on the primary amino acid sequences of the polypeptides by using GOR IV software from the Expasy website (http://www.au.expasy). Moreover, the simulation of the secondary structure of proteins was performed on the I-TASSER-MTD server (multidomain Iterative Threading ASSEmbly Refinement) platform using a hierarchical protocol to predict structures and functions of multidomain (MTD) proteins (https://zhanggroup.org/I-TASSER-MTD/). This protocol predicts the domain boundaries based on the deep-learning contact-map prediction and multiple threading alignments. The individual domain models are assembled into a full-length structure under the guidance of quaternary structural templates and deep-learning distance profiles. The output of the I-TASSER-MTD server includes up to five full-length atomic models (ranked based on the total energy), estimated accuracy of the predicted models (including a confidence score of all models, and root-mean-square deviation (RMSD) for the first model), predicted secondary structures, and predicted solvent accessibility.

#### 2.2.2 Turbidimetric Analysis

The turbidity of UELP and HELP samples was measured as absorbance at λ = 350 nm in the range of 15–50 °C at a heating/cooling scan rate of 0.5 °C·min^–1^ on a Jenway 6300 spectrophotometer. The turbidity was compared to a calibrated zero absorbance measured on the filtered solvent as a blank. Data were fitted by using a Boltzmann sigmoidal function. The inverse transition temperature (*T*_t_) was obtained as the temperature corresponding to 50% of the maximum absorbance value. Purified proteins were dissolved to a final concentration of 2 mg/mL in 10 mM Tris/HCl buffer at pH = 8.0 (Tris) without and with 0.15 M NaCl (Tris/NaCl). Solutions were equilibrated at 4 °C for 16 h before experiments.

#### 2.2.3 Differential Scanning Calorimetry

Thermal properties of lyophilized proteins in solution were evaluated by Differential Scanning Calorimetry (DSC) using a Setaram MicroDSC III DSC model. Stainless steel cells were filled by weight with protein samples (8 mg/mL, in Tris or Tris/NaCl buffer) and then hermetically sealed and equilibrated for 16 h at 4 °C. The calorimeter was pre-equilibrated at 5 °C for 10 min, followed by heating from 5 to 60 °C at a scan rate of 0.5 °C·/ min. The solvent was used as a reference. The inverse *T*_t_ was determined as the peak temperature (*T*_p_). The enthalpy (Δ*H*_t_) and entropy (Δ*S*_t_) of the transition were determined by integration of peak area using in-house-developed graphics software. Lysozyme solution was the calibration standard.

#### 2.2.4 Circular Dichroism

Proteins were dissolved at a concentration of 0.1 mg/mL in Tris/NaCl buffer. CD spectra were recorded at different temperatures in a thermostatic cell from 200 to 500 nm on a Jasco J-710 spectrometer under constant nitrogen flux. Data were reported as the mean molar ellipticity [θ] of the residue (mdeg·cm^2^·dmol^–1^).

#### 2.2.5 Dynamic Light Scattering

The thermoresponsive behavior of human elastin-like polypeptides UELP and HELP was investigated by dynamic light scattering (DLS) using a Malvern Zetasizer Nano ZS instrument (Cambridge, U.K.) equipped with a 4 mV HeNe laser operating at λ = 633 nm, with a measurement angle of 173° backscattering (size diameter range 0.3 nm–10 μm).

DLS was performed on protein solutions at various temperatures and concentrations (2 mg/mL in Tris and Tris/NaCl solutions). The diffusion coefficients *D* and then the hydrodynamic radius *R*_h_ were calculated from intensities (Stokes–Einstein theory) as
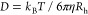
where *k*_B_ is the Boltzmann constant, *T* is the temperature, and η is the viscosity of the solvent. The intensity, volume, and number distributions were calculated by nonlinear least-squares fitting (NLLS, CONTIN algorithm) of the autocorrelation function measured in the experiment. In the case of broader and multimodal distributions, multiexponential fitting was used.

Through DLS analyses, the inverse transition temperature (*T*_t_) and the hydrodynamic diameter (*D*_h_) of UELP and HELP aggregates in Tris and Tris/NaCl solutions were determined on 2 mg/mL biopolymer solutions. DLS analyses were carried out in a temperature range between 10 and 60 °C, with temperature increments of 2 °C and an equilibration time of 180 s for each temperature increase. The temperature at the curve inflection point (i.e., the temperature above which the transition to 100% of a single large particle occurs) was taken as the inverse transition temperature, *T*_t_.

To evaluate the stability of the self-assembled polypeptide aggregates, particle size measurements were made at a fixed temperature above *T*_t_ (40 °C) and repeated every 300 s over a period of 1 h to determine the constancy of the diameters of the particles (Table 1S, Supporting Information).

#### 2.2.6 ^1^H NMR

The temperature-dependent self-assembly of UELP and HELP was also investigated through variable temperature ^1^H NMR spectroscopy. Five milligrams per milliliter biopolymer solutions in D_2_O were prepared and investigated in the 10–60 °C range with consecutive temperature increments of 10 °C, using a Bruker Avance III 400 MHz spectrometer (90° pulse width 7.5 ms, relaxation delay 1 s, acquisition time 1.4 s, and 128 scans).

### 2.3 Cell Culture

The MG-63 and NIH3T3 cell lines were routinely grown in Dulbecco’s modified Eagle’s medium (DMEM, Sigma-Aldrich) supplemented with 2 mM l-glutamine, 100 μg/mL streptomycin, and 100 units/mL penicillin and containing 10% (v/v) heat-inactivated fetal calf serum. Cells were maintained at 37 °C in a saturated humidity atmosphere containing 5% CO_2_ in 25 cm^2^ flasks. To assess the cytocompatibility of recombinant biopolymers, the cells were cultured in a 96-well microplate. Both tissue-culture-treated (TP) and -nontreated (NP) polystyrene plates were used. The wells were filled with 100 μL of a 0.4% (w/v) aqueous solution of each biopolymer that was previously sterilized by 0.22 μm filtration. After overnight incubation at 5 °C, the solution was removed, and the wells were washed two times with 200 μL of sterile water and then air-dried under a sterile hood. Five thousand cells/well were seeded in a final volume of 100 μL. After 24 h, the adhesion assay was performed by crystal violet staining.^14^ Briefly, each well was washed with PBS, and the cells were fixed with 50 μL of 2% (v/v) paraformaldehyde/PBS for 20 min. After two washes, cells were stained with a solution of 0.5% crystal violet in 20% ethanol for 10 min. After extensive washing with water, 50 μL of a 10% acetic acid solution was added to lyse cells, and the microplate was analyzed by an UV/vis plate reader at a wavelength of 600 nm.

## 3 Results and Discussion

### 3.1 Structure of the Recombinant Biopolymers Inspired by Human Elastin

The design of new human elastin homologues started almost two decades ago, and it was initiated with a view to prepare materials with advanced functionality based on components, possibly combining some features of the synthetic polymers, like the very regular structure and the controlled composition, with those of the living organisms, like the biotic origin. Back then, collagen was a well-established paradigm, while elastin and the pentapeptidic motif showing temperature-dependent inverse phase transition behavior was an emerging model.^ ^2b,15^^ At the time, most of the studies were undertaken to adopt a “reductionist approach” since each elastin exon encodes an independent domain with its own structure so that it could be studied and characterized by the use of synthetic peptides resembling its sequence.^16^ However, the opportunity to reiterate the same domain in long chains offered by genetic engineering allowed us to magnify the physicochemical features of a single domain, especially regarding thermoresponsive behavior.^17^

**Figure 1:**
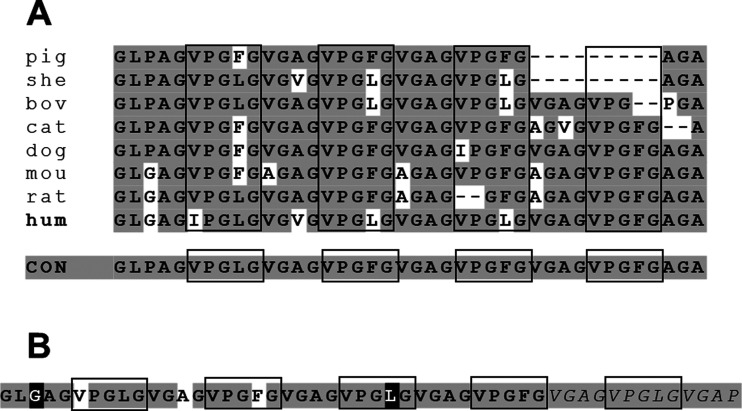
Comparison of part of the exon 26 sequence of elastins from different species. (A) Porcine (XP_020941438.1), ovine (XP_042096308.1), bovine (AAA30505.1), feline (XP_019676153.1), canine (XP_048967017.1), murine (NP_031951.2), rat (NP_036854.1), and human (AAC98395.1) homologues are aligned. In gray are the residues that are the most conserved among these species and that represent the consensus sequence for this region. Boxed, the pentapeptidic motif is followed by a tetrapeptidic block, thus forming the nonapeptidic repeat that characterizes this region. (B) Sequence of UELP hydrophobic domain. Gray, residues corresponding to the consensus; black, residues that are found in the human sequence and were maintained; white, residues that correspond to the consensus and differ from those of the human sequence and that were maintained to enhance the regularity of the repeated sequence; italics, motifs that were repeated to obtain a 50 amino acid domain; boxed, the elastin pentapeptidic repeats.

Thus, following a biomimetic strategy, Bandiera and co-workers focused their attention on the most regularly repeated region of the human elastin homologue. At difference with most of the other elastin-like polypeptides described in the literature at the time, a construct comprising both the cross-linking domains as well as the hydrophobic domains was produced to obtain an ELP biopolymer better resembling the elastin structure. This construct was named HELP (human elastin-like polypeptide).^12^ To characterize the physicochemical properties, a second prototype was also produced^3^ as a reference more closely related to most of the other described ELPs, which were composed of just long stretches of pentapeptidic repeats without any cross-linking domain.^18^ VAPGVG, the hexapeptide-based hydrophobic HELP domain characterizes the primate elastins,^9^ and recently, these sequences were described to improve skin elasticity and reduce wrinkles.^19^ However, the hexapeptidic motif and its permutations are described as matrikines.^20^ Although the HELP turned out to be a valuable component in obtaining hydrogel matrices and a versatile carrier for bioactive domains, this factor may limit, to some extent, the applications of this biopolymer. For this reason, a more accurate analysis of the elastin sequence led to the selection of another monomer to build a construct that overcomes these constraints while maintaining the desired properties. The attention was focused on a regularly repeated as well as much conserved hydrophobic domain among the different organisms in the view of producing a new human-based elastin-like polypeptide with broad compatibility and robust immune tolerance while maintaining the potential as a carrier fusion partner. Aligning several vertebrate elastin amino acid sequences, a highly conserved region is observed, corresponding to part of the exon 26 of the human homologue, which is shown in Figure 1.

**Figure 2:**
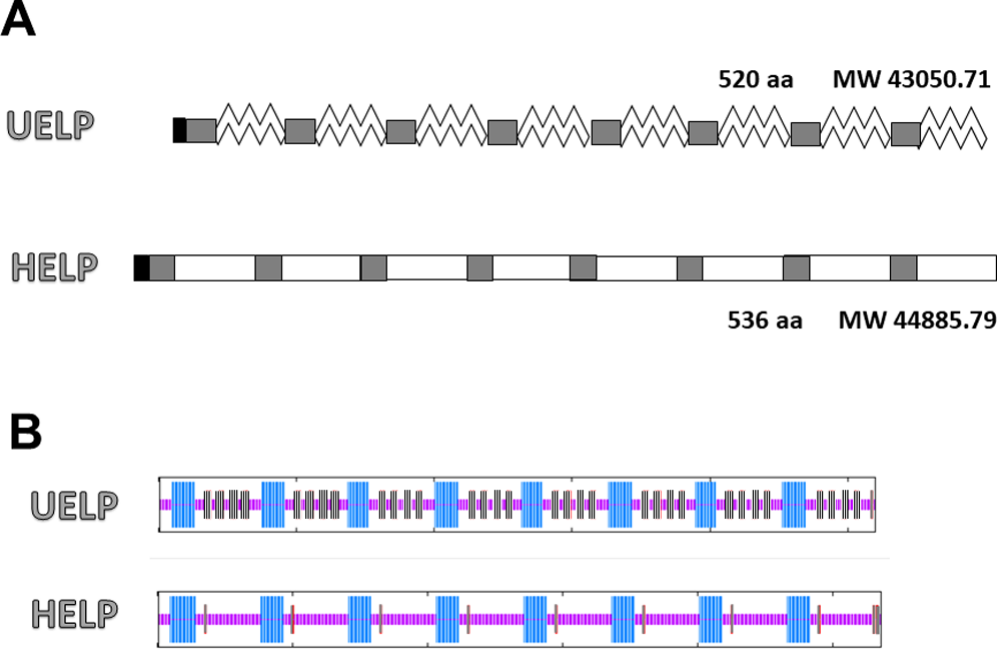
Comparison of the structure of the polypeptides inspired by the elastin human homologue. (A) Schematic representation of the primary structure of the UELP and HELP recombinant proteins. Black, his-tag; gray, cross-linking domains; and white, hydrophobic elastin-like domains. (B) Prediction of the secondary structure of the two biopolymers obtained by I- TASSER simulation. Purple, coil; light blue, helix; and gray, β-strand.

Comparing the sequences, a consensus of 40 amino acids, differing in only five positions with respect to the human sequence, can be outlined, evidencing a nonapeptidic repeat composed of the pentapeptidic, VPGL/FG, and the tetrapeptidic, L/VGAG, motifs (Figure 1A). Interestingly, exon 26 was described to have a dominant role in the temperature-driven self-assembly of elastin.^21^ On this basis, a 50 amino acid repeated sequence identical to the human one except for three positions and one additional nonapeptidic repeat was designed, maintaining the same length of the HELP hydrophobic domain (Figures 1B and 1S). Adopting the same sequence of the HELP cross-linking domains, a new gene that was named “universal” ELP (UELP) with eight reiterated monomers and a length comparable to that of HELP was assembled. In Figure 2A, the schematic primary structures of the two recombinant biopolymers derived from human elastin are compared. They represent a system that allows the amino acid sequence (Figure 1S, Supporting Information) to be correlated with the behavior of the biopolymer as well as with the features of the derived materials and with any biological interaction.

### 3.2 Macromolecular Features of UELP

The distribution of secondary structures in the UELP polypeptide was predicted using GOR IV based on the amino acid sequences. The results, compared with those obtained for HELP, are shown in Figure 2B and Table 1.

**Table 1:** Comparison of the Main Parameters and Distribution of Secondary Structures of UELP and HELP Biopolymers as Predicted Using GOR IV Based on Amino Acid Sequences

		pI	a.a.	Mw	% polar a.a.	% charged a.a.	α %	β %	rc %
UELP	theor	11.7	520	43050	2	4.5	25	24	51
	CD						17	23	60
HELP	theor	11.7	536	44885	2	4.3	26	4	70
	CD						29	10	61

An average α-helix content of 25% for the UELP sequence, very close to the corresponding value for the HELP one, was predicted since, in both biopolymers, the polyalanine stretch is present in the cross-linking domains (Figure 1S). Based on the same calculations, the hydrophobic domains of UELP were predicted to have a mixed, partially disordered structure consisting of 24% β-sheet and 51% random coil regions. The β-sheet fraction of the UELP sequence is significantly higher than that calculated for HELP (4%), which rather possesses a higher fraction of random coil sequences (70 vs 51% of UELP). For both biopolymers, it was predicted that β-sheets occur only in the hydrophobic regions (gray fractions in Figure 2B).

**Figure 3:**
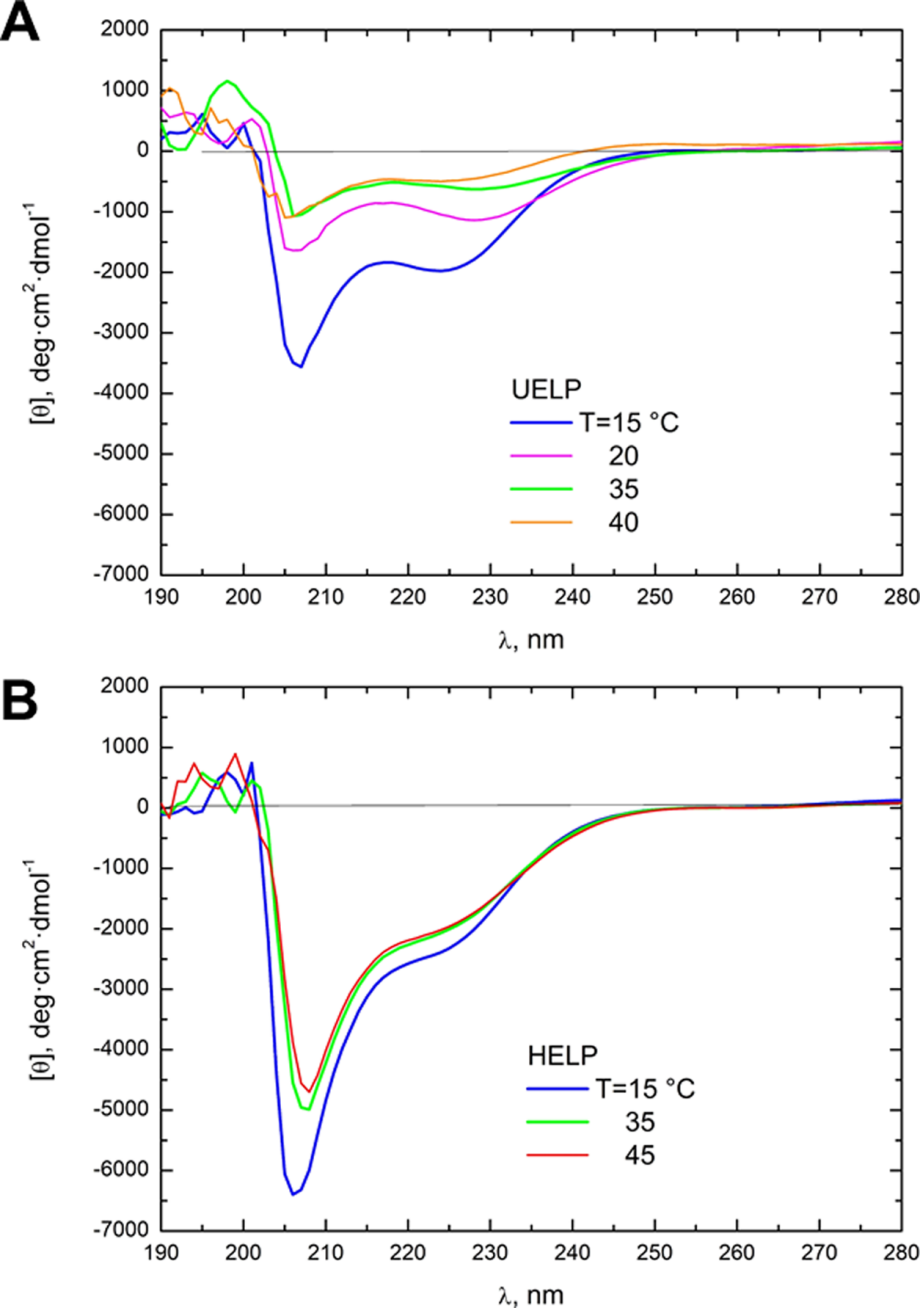
CD spectroscopic analysis of the two elastin-inspired polypeptides UELP (A) and HELP (B) at a concentration of 0.1 mg/mL as a function of temperature: blue line: 15 °C; purple line: 20 °C; green line: 35 °C; orange line: 40 °C; and red line: 45 °C.

Table 1 also shows the distributions of secondary structures for the UELP and HELP biopolymers obtained by deconvolving the spectra of CD measured below the *T*_t_ (Figure 3A,B, blue line), showing consistency between theoretical and experimental data.^22^ Typical negative bands around 200 and 222 nm (ππ* and nπ* transitions, respectively) were observed. The difference between UELP and HELP in the CD signal, mainly around λ = 207 nm (Figure 3), is likely due to the large positive contribution of the β-structure/β-turns domains of the UELP sequence compared to HELP (Table 1), which resulted in a band with a less negative value (cf. Figure 3A with 3B, blue lines). Interestingly, the UELP biopolymer spectra showed a marked dependence on temperature (Figure 3A) with a significant increase of [θ] above the *T*_t_ temperature (>20 °C). This is likely due to the stabilization of the β-structure assembly after the water removal. On the contrary, this trend is not evident for the HELP biopolymer since, increasing the temperature, the CD spectra remained relatively constant, suggesting a predominantly random coiled structure of the hydrophobic domain (Figure 3B).

**Figure 4:**
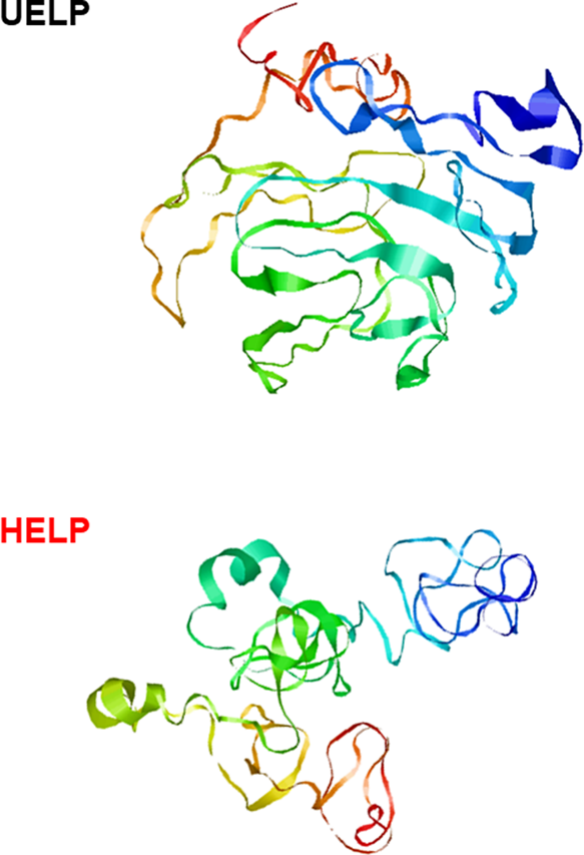
Model of the minimized secondary structure of UELP and HELP obtained by the I-TASSER – MTD simulation.

A snapshot of the two UELP and HELP protein structures (Figure 4) was generated using multidomain I-TASSER-MTD algorithms on the online platform server.^23^ The high-quality three-dimensional (3D) model predictions of the proteins were calculated by deep-learning contact-map prediction and multiple threading alignments starting from the primary structure. Figure 4 clearly shows the larger proportion of β sheet domains of UELP compared to the HELP polypeptide, resulting in a more compact structure, as also supported by the calculated average gyration radii, *R*_G_, from the structures obtained in I-TESSER-MTD simulations, which give *R*_G_ = 7.3 and *R*_G_ = 9.0 nm for UELP and HELP, respectively.

### 3.3 Physicochemical Properties of UELP and HELP

#### 3.3.1 Turbidimetric Analysis

**Figure 5:**
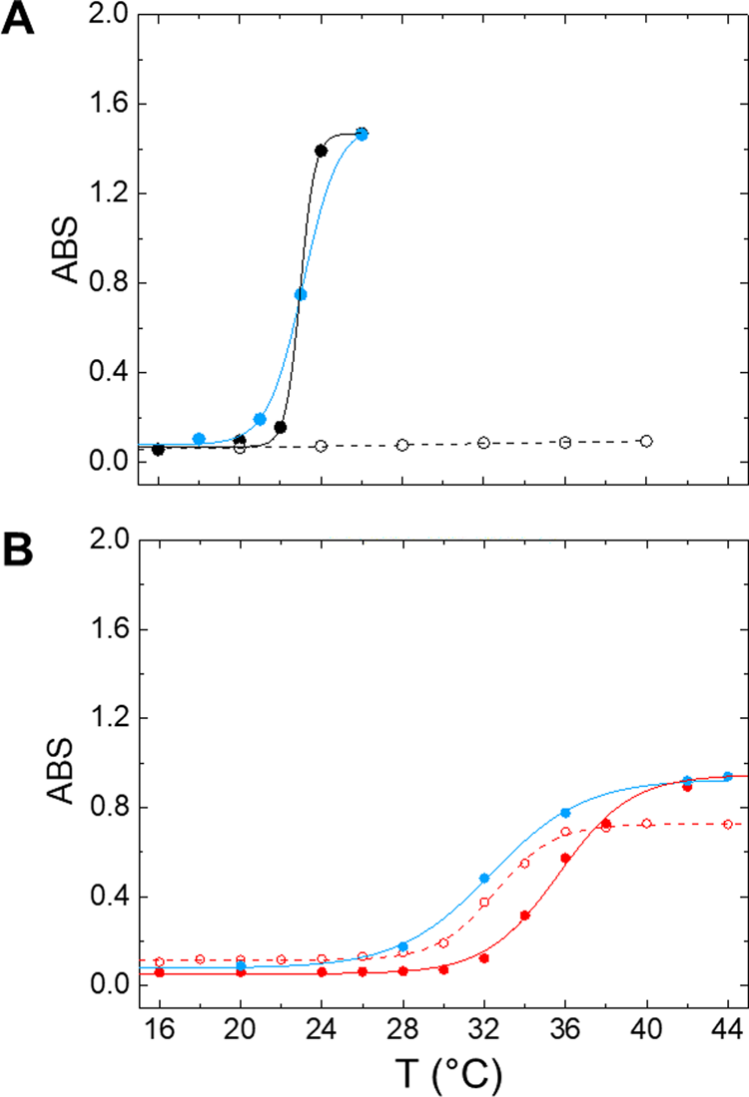
Turbidimetric analysis of the human elastin-derived biopolymers as a function of temperature. UELP (A) and HELP (B) were solved at 2 mg/mL in 10 mM Tris buffer (open symbols) and in Tris/NaCl (solid symbols). Cooling turbidity profiles (in blue) were analyzed in Tris/NaCl buffer.

The inverse thermal transition of UELP in solution was studied by turbidimetric and calorimetric measurements, comparing its behavior with that of the polypeptide HELP in the absence and presence of a nearly physiological salt concentration. It is known that the presence of cross-linking domains among the hydrophobic sequences of elastin strongly influences its thermoresponsive behavior. A near-physiological NaCl concentration is required for optimal coacervation of these types of primary structures.^2b,7,24^ On the other hand, for ELPs, which in most cases do not have cross-linking domains, the addition of salt lowers T_t_, so this condition is exploited for the purification of these polypeptides.^15,18,24b,25^ Thus, salt concentration likely plays an awkward role in modulating the phase transition of polypeptides that have alternating hydrophobic and cross-linking domains in their sequence, mimicking the primary structure of elastin. The hydrophobic folding and self-assembly processes of UELP and HELP were followed at a specific temperature scanning rate, as described in Section 2. Figure 5 shows the results of the turbidimetric analysis of UELP compared to the biopolymer HELP, which was previously characterized.^7^

Strikingly, in the absence of salt, the 2 mg/mL UELP biopolymer solution (Figure 5A, open symbols) shows a negligible turbidity variation. The *T*_t_ of about 27 °C was determined by fitting the transition curve with a Boltzmann sigmoidal function. On the other hand, the HELP sample shows an increase in turbidity of the solution with a *T*_t_ of 32 °C under the same conditions (Figure 5B, open symbols).

The addition of 0.15 M NaCl to the UELP biopolymer solutions resulted in a significant and sharp increase in turbidity at a *T*_t_ of approximately 22 °C (Figure 5A, filled symbols), indicating full recovery of the transition phase property. In the case of HELP, the addition of a near-physiological salt concentration tended to increase the *T*_t_ to about 35 °C (Figure 5B, filled circles).

However, this is consistent with our previous observation on dilute solutions of the biopolymer HELP.^7^ A polypeptide consisting of the same HELP hydrophobic hexapeptidic sequences but lacking the cross-linking domains showed significantly higher *T*_t_ with respect to HELP and was not affected by the addition of a near-physiological salt concentration.^7^ In contrast, the addition of the same salt concentration to the HELP solution resulted in an increase in *T*_t_, suggesting that HELP, once the effect of the presence of the cross-linking domains is attenuated by a near-physiological salt concentration, tends toward the *T*_t_ of the sequence without the cross-linking domains.^7^

The behavior of the UELP biopolymer was markedly different from that described above for HELP, suggesting that the presence of the cross-linking domains alternating with the elastin-like regions based on the nonapeptide repeats of exon 26 had a dramatic effect that nearly abolished the ability of UELP to phase transition. However, the addition of salt at near-physiological concentrations fully restored the thermoresponsive behavior of the UELP biopolymer, which exhibited a sharper transition at a much lower *T*_t_ with respect to that of HELP, confirming that this salt concentration is essential to avoid hampering the temperature transition process of the elastin-like sequences in the presence of the cross-linking domains. The reversibility of the phase transition of UELP and HELP was analyzed in the presence of a near-physiological salt concentration by cooling the samples after the transition. A clear difference between the two biopolymers can also be seen in this process (Figure 5A,B, see the blue lines). In the case of UELP, the curve obtained by cooling almost overlaps with the aggregation curve, while HELP heating and cooling ramps lead to two different, less steep curves that exhibit some hysteresis, suggesting a more stable supramolecular configuration as a function of temperature.

Taken together, these results indicate different self-assembly behaviors of the two biopolymers. The sharper transition of UELP and its prompt reversal compared with the slower HELP turbidity increase with hysteresis during cooling suggested two different aggregation and dissolution mechanisms. The observed different values of the average gyration radii calculated above, which are lower for UELP than for HELP suggest different compaction capacities of the two different hydrophobic sequences. On the other hand, the presence of the cross-linking domains in the biopolymers may also contribute to explaining the different hysteresis observed. Thus, in addition to the interactions among the hydrophobic elastin-like domains, an interplay among the cross-linking domains may be expected.^26^ In the case of UELP, the hydrophobic sequences derived from the exon 26 are optimized to strongly promote the self–assembly to a more compact structure,^27^ likely overcoming all other possible interactions. Conversely, the delayed HELP coacervation process may allow further interactions beyond the hydrophobic aggregation,^26^ leading to a more stable final configuration.

#### 3.3.2 Differential Scanning Calorimetry

DSC experiments were performed to compare and further verify the inverse phase transition properties of UELP and HELP biopolymers. The results are shown in Table 2. The measurements were performed under the same conditions as the turbidimetric analyses, and the behavior of the biopolymers was analyzed in the same Tris buffer solution with and without 0.15 M NaCl. Except for UELP in the absence of salt, an endothermic asymmetric peak was always observed.

**Table 2:** Thermodynamic Results of the DSC Analysis of 8 mg/mL UELP and HELP in 10 mM Tris Buffer, pH = 8, in the Absence and Presence of 0.15 M NaCl

		*T* peak	Δ*H*_tr_ kJ/mol	Δ*S*_tr_ J**/mol K**
UELP	TRIS	ND	ND	ND
	TRIS/NaCl	23	29.0	98
HELP	TRIS	29	198.0	655
	TRIS/NaCl	34	35.0	114

According to the turbidimetric analyses, UELP in the presence of NaCl exhibited the lowest peak *T*_t_ (23 °C) and showed a greater tendency to transition compared to HELP. As previously reported,^7^ Δ*H*_tr_ can be a useful method for studying the relative hydrophobicity of polypeptides because the lower the transition enthalpy, the lower the hydrophobicity of the polypeptide. Prediction from the sequence data showed that UELP and HELP had similar proportions of polar and charged groups (6.5 and 6.3%, respectively, Table 1), resulting in similar Δ*H*_tr_ (29 and 35 kJ/mol, respectively) and Δ*S*_tr_ values (98 and 114 kJ/mol K, respectively), although UELP always had the lowest values, indicating lower hydrophobicity compared with HELP. The DSC data in Table 2 show good agreement between the *T*_t_ values and those obtained by turbidimetric analysis under the same conditions (Figure 5). According to these analyses, the data in Table 2 show a significant difference in *T*_peak_ temperatures between UELP and HELP proteins, probably due to the higher proportion of β-structures in the UELP sequence. It is likely that, although HELP shows a higher hydrophobicity with respect to UELP, this latter has a higher propensity to adopt the β-structure, making it the most efficient in promoting the hydrophobic interactions and the supramolecular assembly.^27,28^

#### 3.3.3 Dynamic Light Scattering Characterization

By using the DLS technique, we measured the hydrodynamic diameters of the biopolymers in solution and the dimensions of the aggregate sizes as a function of temperature. Figure 2S shows the intensity and volume size distribution of the hydrodynamic diameter (*D*_h_) for UELP and HELP at different salt concentrations at 15 °C. The size distribution, determined as the scattering intensity, showed a multimodal pattern over a wide dimensional range, indicating the presence of particles of various sizes, most of which were centered around 10 nm, as confirmed by the volume size distribution (Figure 2S). This indicates that, below the transition temperature, the smallest biopolymer particles were predominant at room temperature, while the proportion of the largest self-assembled particles was low despite the total scattering intensity being the highest.

**Figure 6:**
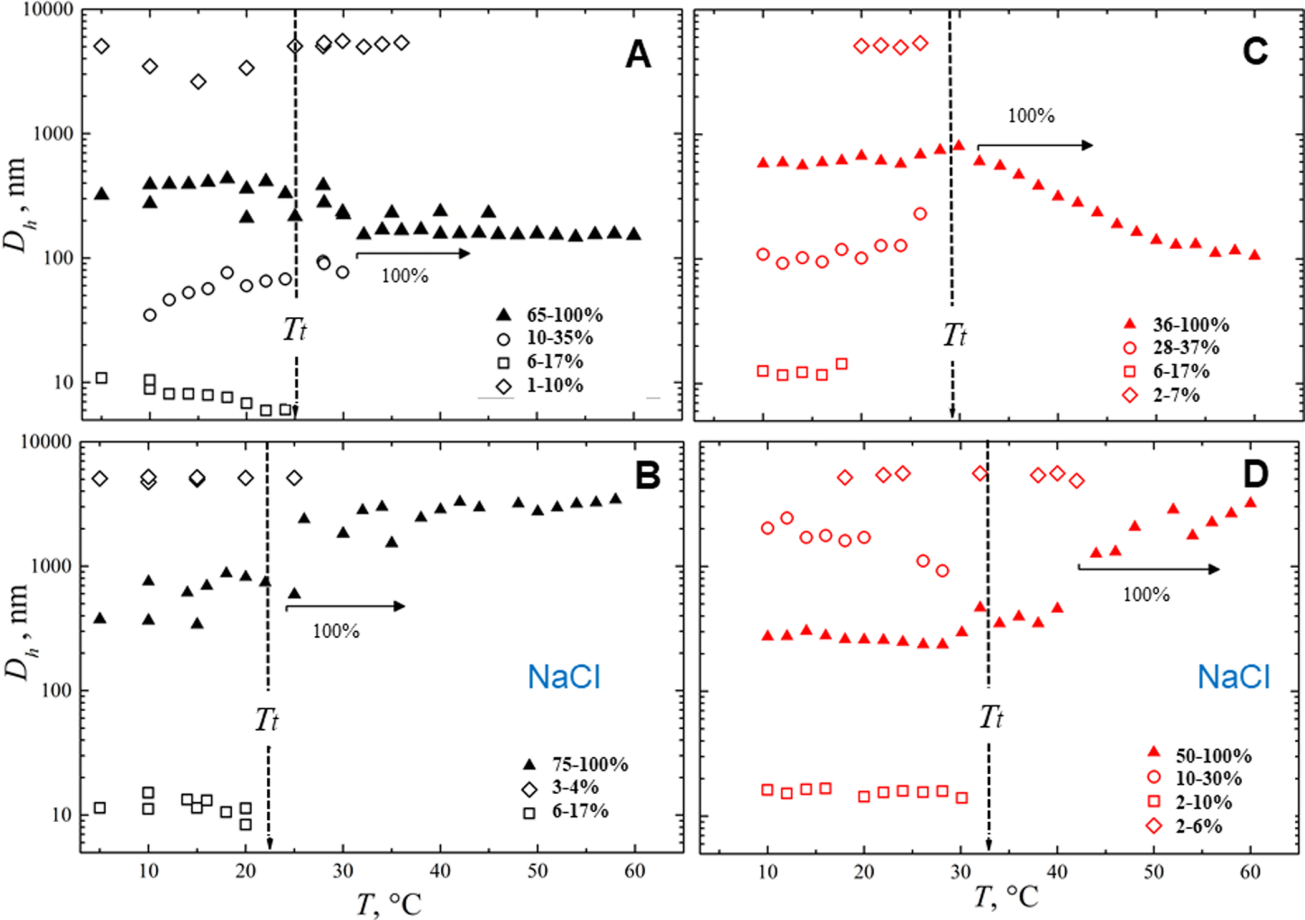
DLS diameters (intensity-based calculated values) for UELP (black symbols) and HELP (red symbols) in 10 mM Tris (A, C, respectively) and in 10 mM Tris/NaCl buffer (B, D) at a concentration of 2 mg/mL as a function of temperature ranging from 10 to 60 °C at a scanning rate of 0.5 °C/min. The vertical dashed bars show the respective *T*_t_ values; the horizontal arrows show the predominant size distribution.

Figure 6 shows the average diameter values, *D*_h_, of UELP (Figures 6A,B, black symbols) and HELP (Figure 6C,D, red symbols) in the absence and presence of a near-physiological NaCl concentration as a function of temperature.

The percentages of the peak areas (Figure 6A–D, in the insets), as well as the particle size values, were determined from scattering intensity distribution. *T*_t_ was determined at the inflection point of the DLS curve for each sample and is evidenced in Figure 6 (vertical dashed bars).

In the absence of salt and below *T*_t_, a multimodal size distribution was observed for both biopolymers at a concentration of 2 mg/mL (Figure 6A,C). A four-modal size distribution (average *D*_h_ of 10, 60, 300, and 3500 nm) was observed for the UELP biopolymer (Figure 6A), with a prevalence (65–100%) of the *D*_h_ = 300 nm-sized particles. Under the same conditions, the HELP biopolymer (Figure 6C) showed a similar four-modal size distribution as well, with the main fraction (36–100%) consisting of particles with a *D*_h_ = 600 nm. Interestingly, although the two biopolymers showed different behavior above the *T*_t_, both exhibited a monomodal particle size distribution, with an average particle size of about 150 nm at the highest temperature studied (60 °C, Figure 6A,C). However, despite the temperature increase, the UELP particle size remained constant (Figure 6A), whereas the HELP particle size gradually decreased with the temperature rise (Figure 6C). In the presence of 0.15 M NaCl and below *T*_t_, the UELP biopolymer showed a three-modal particle size distribution (Figure 6B), with a prominent fraction of *D*_h_ = 600 nm (75%, filled triangles) and two smaller fractions of *D*_h_ = 10–15 nm (6–17%, open squares) and *D*_h_ = 5000 nm (3–4%, open diamonds). Above *T*_t_, again, a monomodal particle size distribution was observed, with a tendency to stabilize aggregates with a *D*_h_ of about 3000 nm (Figure 6B, filled triangles with 100% scattered light). Below *T*_t_, the HELP biopolymer (Figure 6D) showed a four-modal distribution with a main fraction (about 50%, filled triangles) with a *D*_h_ of 250 nm. Above the *T*_t_, a further temperature increase resulted in a monomodal particle size distribution with a gradually increasing *D*_h_ up to about 3000 nm (Figure 6D, filled triangles, 100% of scattered light).

These results show that in the absence of salt and above *T*_t_, the HELP sample has a tendency to gradually decrease in particle diameter, suggesting a change from expanded to contracted structures as a function of temperature as previously described for these hexapeptidic sequences.^24b^ In contrast, under these conditions, the UELP particle size stabilized around a value that remained constant despite the temperature increase (compare Figure 6A with 6C), suggesting prompt and optimized particle assembly. It can be surmised that for the HELP biopolymer, the structural transition occurred gradually over a temperature range of 30 °C (Figure 6C), which could be due to the higher chain flexibility of the HELP compared to the UELP biopolymer. This is also confirmed by the secondary structure analysis (Figure 2B and Table 1), which shows a higher proportion of random coil sequences in HELP compared with UELP (70 and 51%, respectively). HELP may, therefore, undergo a progressive molecular collapse associated with a realignment of water molecules and a restructuring of hydrogen bonding networks (i.e., peptide–peptide hydrogen bonds replace water–water hydrogen bonds in the nearest solvation shells), gradually displacing water from the hydrophobic moiety and leading to a decrease in particle size.^24b^ On the other hand, a significant presence of β-domains in the hydrophobic sequences of UELP is expected (Figure 2B and Table 1), and this likely leads to a more efficient structural collapse process in the local secondary structure and to a rapid rearrangement of water once the critical *T*_t_ threshold is reached.^29^

**Figure 7:**
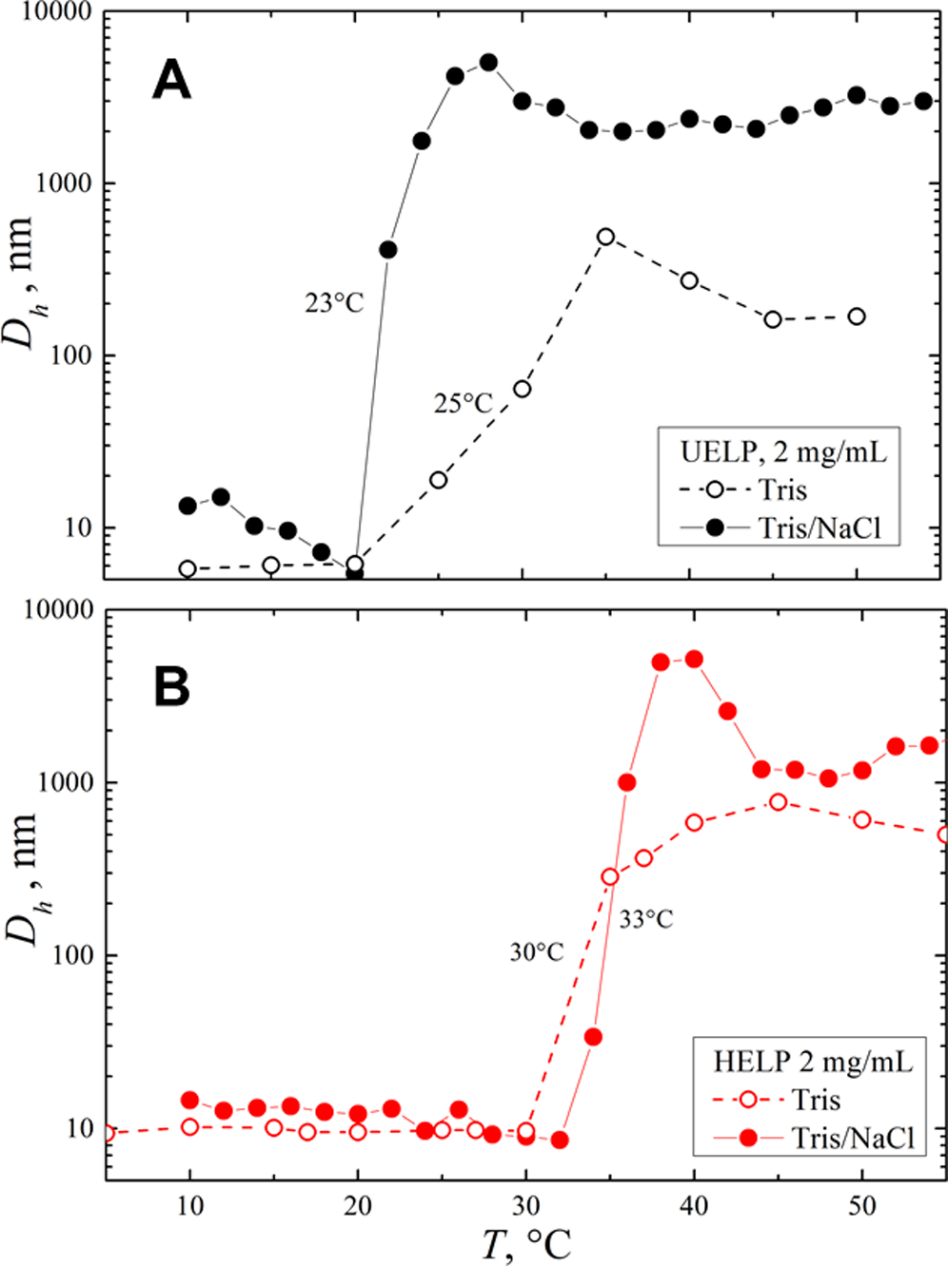
DLS diameter (volume-based calculated values) as a function of temperature. Calculated values of particle distribution in Tris (open symbols) and Tris/NaCl buffer (filled symbols) at a concentration of 2 mg/mL for UELP (A) and for HELP (B).

According to our previous observations and the results of turbidimetric analyses, the decrease in *T*_t_ of UELP upon addition of salt (Figure 7A) and the previously observed increase in *T*_t_ of HELP upon addition of salt (Figure 7B) confirmed the expected critical role of physiological salt concentration in restoring the thermoresponsive properties of elastin-like sequences when inserted between cross-linking domains.

The effect of salt addition not only masks the effect of cross-linking domains but also leads to a different interaction between ions, the hydrophobic thermoresponsive sequence, and water molecules in the nearest hydration shells. Ions diffusing into the nearest hydration shell of the polypeptide can interact strongly with the peptide chain and facilitate the structural folding of the hydrophobic domain.^30^ In addition, the ions can disrupt the hydrogen-bonded water network around the protein and promote the formation of hydrogen bonds within the hydrophobic sequence moiety while displacing solvation water molecules from the nearest hydration shell. In the presence of salt and above the *T*_t_, both biopolymers showed the ability to form particles with larger dimensions than in the absence of salt. Above *T*_t_, the UELP biopolymer, during the temperature increase, showed a constant particle size with a large *D*_h_ of about 3500 nm during the temperature increase (Figure 6B), while the particles of HELP showed a gradual trend of increasing diameter from about 300 nm up to 4000 nm under the same conditions (Figure 6D), again indicating greater chain flexibility (higher entropy) requiring higher temperature to stabilize the particle size. Figure 7 shows the particle diameters determined by DLS as the percent particle number distribution (N%) for the two biopolymers at a concentration of 2 mg/mL. In the absence of NaCl and below *T*_t_, the particle diameters for the biopolymers were about 6 and 10 nm for UELP and HELP, respectively (Figure 7, open symbols), which most likely corresponds to a single chain size in solution. It is interesting to note that the values of the hydrodynamic diameter *D*_h_, which are calculated from *R*_G_(31) using the equation
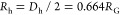
resulted in a *D*_h_ of 9.7 nm and 12.0 nm for UELP and HELP, respectively, thus showing values in agreement with the DLS diameters measured for the temperature below the *T*_t_ (Figure 7). In the absence of NaCl and above *T*_t_, the UELP biopolymer formed particles that stabilized at a *D*_h_ greater than 200 nm, while HELP formed larger particles 500–800 nm in diameter. The addition of salt at near-physiological concentrations had a remarkable effect on the diameter of UELP particles, which promptly increased from a value of about 6 nm in the absence of salt and near *T*_t_ (Figure 7A, open symbols) to about 5000 nm in the presence of NaCl (Figure 7A, filled symbols). This value, which stabilizes as a function of temperature at about 3000 nm, is significantly larger than the value observed in the absence of salt (above 200 nm). In the presence of salt, HELP also showed a remarkable change in particle diameter around T_t_, shifting from 10 to 15 to 5000 nm (Figure 7B, filled symbols) but stabilizing at about 1200–1700 nm as a function of temperature. However, in the case of HELP, particle sizes remained comparable in the presence and absence of salt (Figure 7B, see the filled and open symbols).

#### 3.3.4 ^1^H NMR Spectroscopy

**Figure 8:**
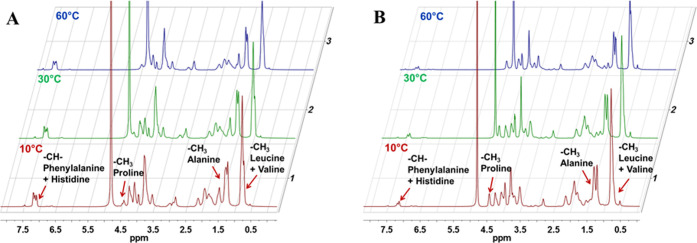
Overlay of ^^1^^H NMR spectra of UELP (A) and HELP (B) in D__2__O (5 mg/mL) at 10, 30, and 60 °C. Arrows indicate the resonance peaks of the following amino acid residues: leucine + valine, proline, alanine, and phenylalanine + histidine.

The arrangement of UELP and HELP biopolymers in solution was studied by ^1^H NMR spectroscopy in D_2_O to evaluate differences in the polypeptide supramolecular arrangements occurring upon thermally induced coacervation. Figure 8 shows, as an example, the NMR spectra of HELP and UELP in a D_2_O solvent. The characteristic resonances of some protons of the amino acid residues at 10 °C, i.e., under the conditions of maximum solubility, are shown in Table 3. In particular, the signals of −CH_3_ protons of leucine and valine at 0.76 ppm and −CH_3_ of alanine were clearly visible (Figure 8).

**Table 3:** Total Number of Amino Acid Residues (n res) and Relative Protons (nH) of UELP and HELP^a^

	UELP				HELP			
	theoretical		NMR		theoretical		NMR	
	n res	nH	δ (ppm)	nH	n res	nH	δ (ppm)	nH
L + V	109	654	0.76	658	129	774	0.76	860
A	130	390	1.03–1.38	412	159	477	0.97–1,46	590
P	48	48	4.40	43	72	72	4.42	75
F + H	29	127	6.55–7.54	127	14	52	6.57–7.55	52

a Chemical shifts (δ) and proton number (nH) of both biopolymers determined by NMR analysis.

**Figure 9:**
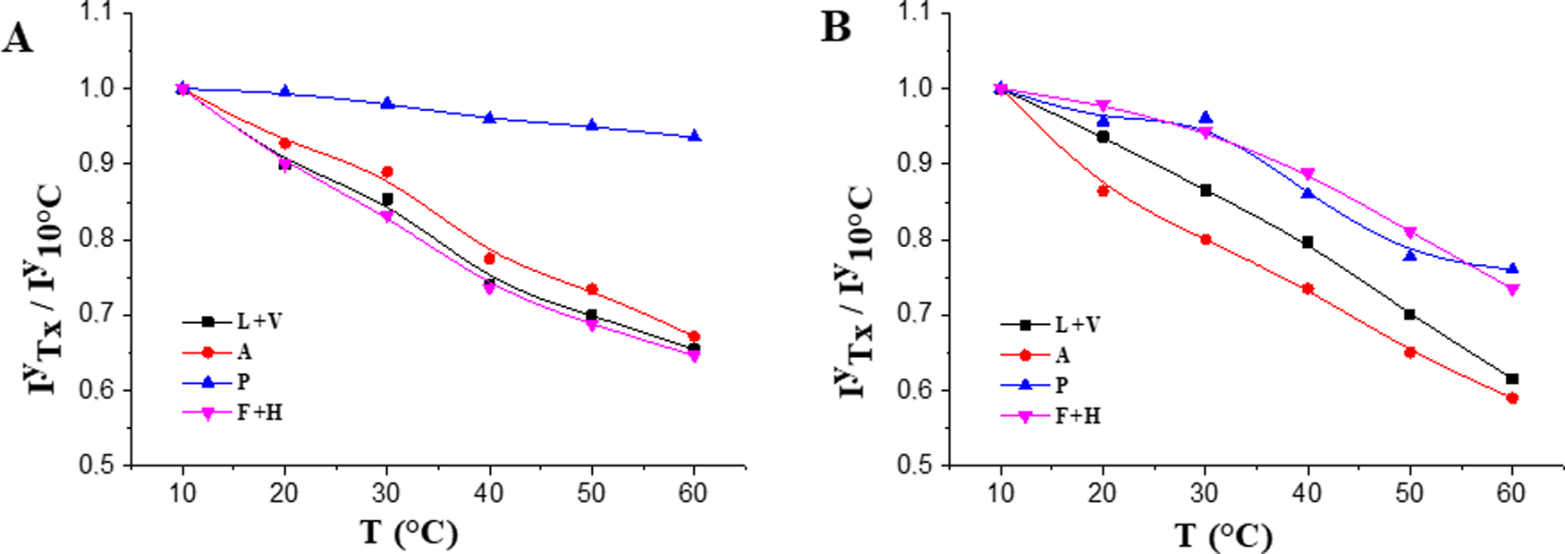
*I*^y^*_Tx_*/*I*_10 °C_^y^ as a function of temperature for UELP (A) and HELP (B), as determined by ^1^H NMR in D_2_O (5 mg/mL) in the range between 10 and 60 °C.

The formation of supramolecular aggregates by thermally induced self-assembly was studied by ^1^H NMR at a variable temperature. Upon heating, a significant decrease in the resonance peak areas was observed (Figure 8), along with their downward shift. The latter is clearly visible in Figure 3S, where the chemical shift of the resonance peak was plotted as a function of the temperature for each amino acid residue. A nearly linear trend with an increasing temperature was observed for all proton groups, suggesting that the increase in temperature weakens the hydrogen interactions between the polar amino acid groups of the polypeptide and the water and decreases the solvation and electron shielding at the hydrogen nuclei.^32^ In addition, the ratio of the absolute integral at a given temperature (*T*_*x*_) to the integral at 10 °C (*I*_*Tx*_^y^/*I*_10 °C_^y^) was calculated for each proton resonance peak and plotted as a function of temperature (Figure 9). It can be noticed that most of the peak integrals gradually decreased with temperature increase, with no evidence of sharp transitions associated with the occurrence of *T*_t_. A similar trend in UELP and HELP integrals was observed for the proton peaks of alanine, leucine, and valine, suggesting that these residues exhibit progressively stronger hydrophobic interactions upon heating, reaching a signal decrease of about 33–41% at 60 °C, with a slightly larger decrease for residues in HELP than the analogues in UELP.

Notably, the greatest decrease was observed for UELP phenylalanine and histidine signals (Figure 9A), which may be attributed to the aromatic side chains and the higher proportion of phenylalanine residues in this polypeptide. Interestingly, a striking trend was observed for proline protons. In fact, the intensity ratio of UELP prolines in Figure 9A decreased by only 5% at 60 °C, indicating that strong interactions with water molecules persist when particle aggregation occurs. Since prolines are known to be present mainly in the β-sheet structures, which are generally involved in self-association and subsequent coacervation,^28^ this behavior suggests that the UELP β-sheet structures are still stable and solvated after polypeptide self-assembly. In this context, the observed slight decrease in the level of the proline signal is attributed to the rearrangement of the proline residues not involved in the β-sheet structures after self-assembly.

A different trend in the proline signal intensity was observed for HELP. Figure 9B shows a decrease in proline intensity (up to 24%) as a function of temperature, suggesting that in this case, the proline residues are actively involved in the coacervation process of HELP, whereupon they are buried in the hydrophobic moiety. As previously reported,^24b^ temperature-driven coacervation of highly hydrophobic elastin-like proteins may occur by decreasing the hydrodynamic radius and expelling water to reduce the hydrophobic-solvent interaction as the temperature increases. From this point of view, proline, as well as other residues belonging to the hydrophobic domains of HELP (alanine, valine, and leucine), could be involved in these temperature-driven structural changes so that their peaks show a larger decrease in HELP.

In summary, NMR analysis is consistent with previous analyses and highlights a different thermally driven coacervation mechanism for the two biopolymers due to the peculiar local secondary structure of their hydrophobic sequences.

### 3.4 Cytocompatibility Evaluation

**Figure 10:**
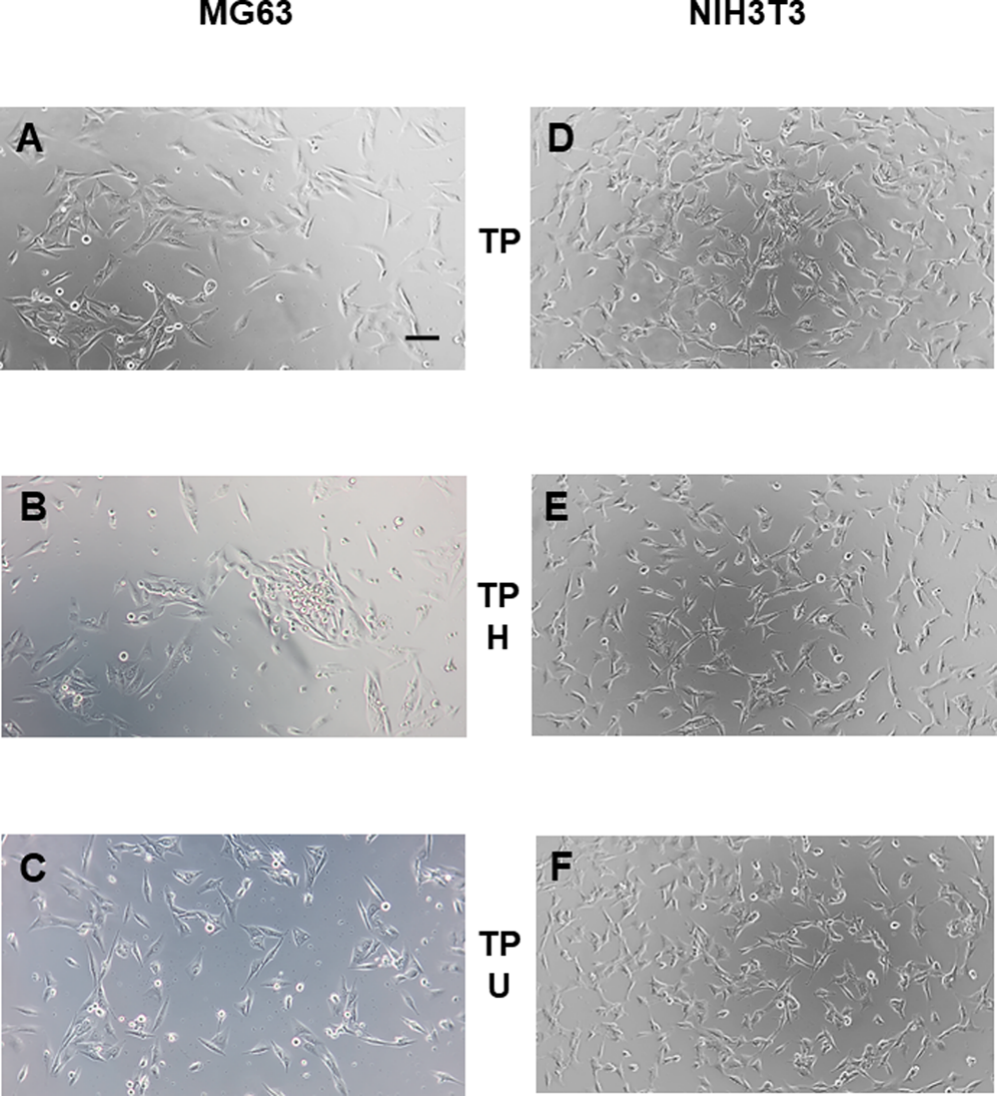
Representative phase-contrast images of cell cultures on coated and uncoated tissue-culture polystyrene wells (TP). MG-63 and NIH3T3 cell lines of human and murine origin, respectively, were seeded on uncoated TP wells (panels A and D) and on TP wells coated with HELP (TP -H, panels B and E) and UELP (TP -U, panels C and F) and grown under standard conditions. Images were acquired 24 h after seeding. The bar is 100 μm.

**Figure 11:**
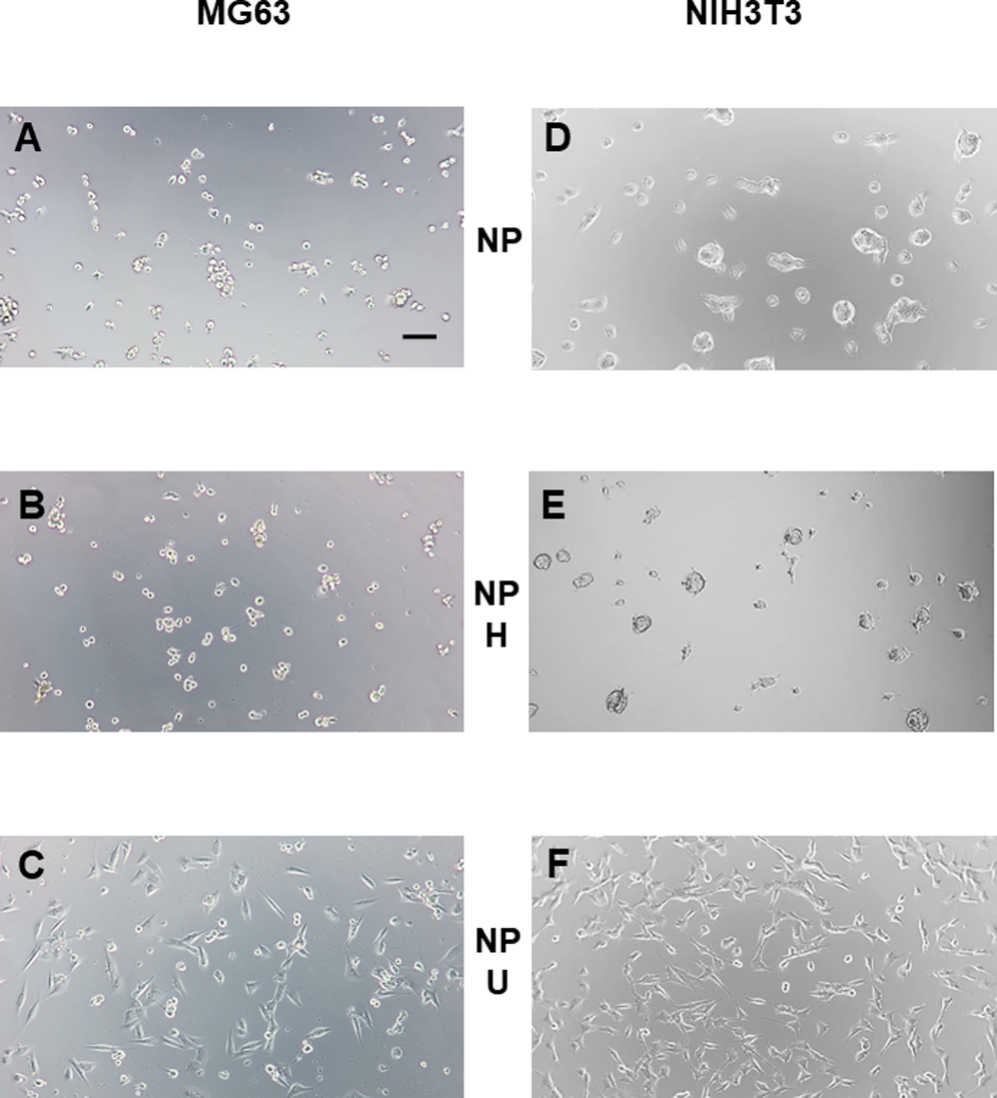
Representative phase-contrast images of cell cultures on coated and uncoated nontissue culture polystyrene (NP). MG-63 and NIH3T3 cell lines of human and murine origin, respectively, were seeded on the uncoated NP wells (panels A and D) and on the NP wells coated with HELP (NP -H, panels B and E) and UELP (NP -U, panels C and F) and grown under standard conditions. Images were acquired 24 h after seeding. The bar is 100 μm.

HELP biopolymers have been used as substrates for the culture of human cells of various origins. However, it was found that in some cases, cell adhesion after 24 h varied depending on the cell line used and the thickness of the biopolymers on the surface.^6,12,33^ To compare the cell adhesion ability of the new UELP versus the biopolymer HELP, tissue-culture-treated polystyrene (TP) was coated with each biopolymer by adsorption, as described in Section 2. MG-63 human osteoblast-like cells and NIH3T3 mouse fibroblasts were seeded, and after 24 h, no significant differences in adhesion were observed for either cell line on each biopolymer coating compared with the TP surface (Figure 10). Interestingly, when the same coating procedure was performed on an untreated polystyrene microtiter plate (NP), a notable difference in adhesion was seen for both cell lines after 24 h (Figure 11). The cells were not able to adhere to the uncoated surface NP as expected (Figure 11, panels A and D). Cells seeded onto the HELP-coated surface NP also behaved similarly to cells observed on the uncoated control surface NP: They showed a rounded morphology and formed small aggregates, suggesting poor adhesion to the surface at this time point (Figure 11, panels B and E). In contrast, cell adhesion on the UELP-coated surfaces of NP in both cell lines was comparable to that observed in the TP control (compare Figure 11, panels C and F, with Figure 10, panels A and D).

The crystal violet adhesion test confirmed this observation (Figure 4S) and confirmed a promoting effect on cell adhesion. However, after a longer time, e.g., 48 or 72 h, depending on the cell line, cells were able to cover all coated surfaces of NP and show their characteristic morphology, indicating that the presence of the biopolymers has no toxic effect (data not shown).

In addition, coatings were prepared by decreasing the concentration of the biopolymer solutions used for adsorption on NP. No significant difference was observed for the HELP-coated surfaces, whereas a dose-dependent cell response was observed on UELP coatings. This effect correlated with the amount of UELP biopolymer present in the solution used to prepare the coatings. Cell metabolic activity was evaluated 24 h after seeding by the WST-1 assay (Figures 5S and 6S). This analysis showed that the UELP and HELP coatings have no toxic effect on both cell lines, and the cell adhesion-promoting effect of UELP was confirmed (see the Supporting Information).

Remarkably, UELP and HELP have the same structure with alternating elastin-like and cross-linking domains, the same length, and very similar composition, whereas only the amino acid sequence of the elastin-like domain differs (Figure 1). The data presented here suggest that the sequence of the elastin-like domain, the sequence inspired by exon 26 rather than human exon 24, promotes the adhesion of cells of different origins to nonadhesive NP surfaces. Although tropoelastin has long been considered an unstructured protein, the 3D shape of the human homologue has been described using an unconventional approach.^34^ According to this model, the region encoded by exon 26 was found to have the highest protease susceptibility, indicating that this sequence is exposed.^34^ Thus, this region could also be readily accessible to cells organized in the extracellular matrix of the tissue. This could be one of the possible explanations for why this highly conserved region was well tolerated by the cells and may even represent a point of cell attachment. On the other hand, the exon 24-derived region is also exposed, being located in the so-called “spur” of the tropoelastin structure.^34^ However, it can be considered that the sequence of this region, being peculiar to the human, and more in general, primate homologue and also having a recognized signaling role,^20^ is less likely to represent a stable adhesion point for the cells within the extracellular matrix.

## 4 Conclusions

A new ELP sequence was designed and fabricated to improve cyto- and tissue compatibility and to extend the feasibility of this class of recombinant biopolymers and derived materials to the veterinary field while maintaining typical thermoresponsive properties. The new UELP construct was successfully prepared, and the expression product was characterized, focusing on the comparison of its physicochemical behavior to that of the previously described biopolymer HELP.

Our study highlights the effect of elastin-like sequences mimicking the different hydrophobic domains of human elastin interspersed with the cross-linking domains, leading to the realization of biomimetic elastins that, in addition to phase transition properties, exhibit significantly different features in thermoresponsive behavior. These results indicate that our recombinant platform is a valuable tool to further elucidate the physicochemical properties of elastin and related sequences.

The new UELP polypeptide showed an improved ability to promote the adhesion of cells from different origins to nonadhesive surfaces compared to the biopolymer HELP. Overall, our system, which ensures tight control over the bioinspired structure of the polypeptides, provides a powerful means to analyze how the extracellular environment can influence and potentially control cell response.

These results demonstrate that our approach can lead to the production of biomimetic components that have at least two valuable aspects that can be exploited. One relates to their application for the development of biocompatible materials with advanced functionality, and the other relates to their use as specific and customizable tools to study and elucidate the interaction at the interface of materials and biological systems at the molecular level.

## Supporting Information Available

Primary structure of the proteins described in the paper; size distribution of the apparent hydrodynamic diameter of the biopolymers’ particles; chemical shift of resonance peaks as a function of temperature; cell adhesion and viability assays (PDF)
