# Supplementary material for: Physicochemical Characterization of a Biomimetic, Elastin-Inspired Polypeptide with Enhanced Thermoresponsive Properties and Improved Cell Adhesion
Source: Biomacromolecules. 2023 Oct 27;24(11):5277–89. doi: 10.1021/acs.biomac.3c00782 (PMC10647011; doi:10.1021/acs.biomac.3c00782)
Supplement: Supplementary file 1 — bm3c00782_si_001.pdf [file bm3c00782_si_001.pdf]

Physico-chemical characterization of a biomimetic, elastin inspired polypeptide with enhanced thermo-responsive properties and improved cell adhesion.

Antonella Bandiera[*1], Laura Colomina - Alfaro[1], Paola Sist[1], Giovanna Gomez d'Ayala[2*], Federica Zuppardi[2], Pierfrancesco Cerruti[2], Ovidio Catanzano[2], Sabina Passamonti[1], and Ranieri Urbani[3]*

[1]*Department of Life Sciences, University of Trieste, via L. Giorgieri, 1, 34127 Trieste, Italy.*

[2]*Institute for Polymers, Composites and Biomaterials (IPCB-CNR), Via Campi Flegrei 34, 80078 Pozzuoli, NA, Italy.*

[3]*Department of Chemical and Pharmaceutical Sciences, University of Trieste, via L. Giorgieri, 1, 34127 Trieste, Italy.*

		T_t (°C)	D_h (T= 40°C) /nm
**UELP**	**TRIS**	25	178
	**TRIS/NaCl**	23	1930
**HELP**	**TRIS**	29	204
	**TRIS/NaCl**	33	439

TABLE 1S

T_t and hydrodynamic diameter (D_h) of 2 mg/mL solutions at 40°C. To verify the particle diameter stability, the measurements were repeated at 1 hour intervals at constant temperature.

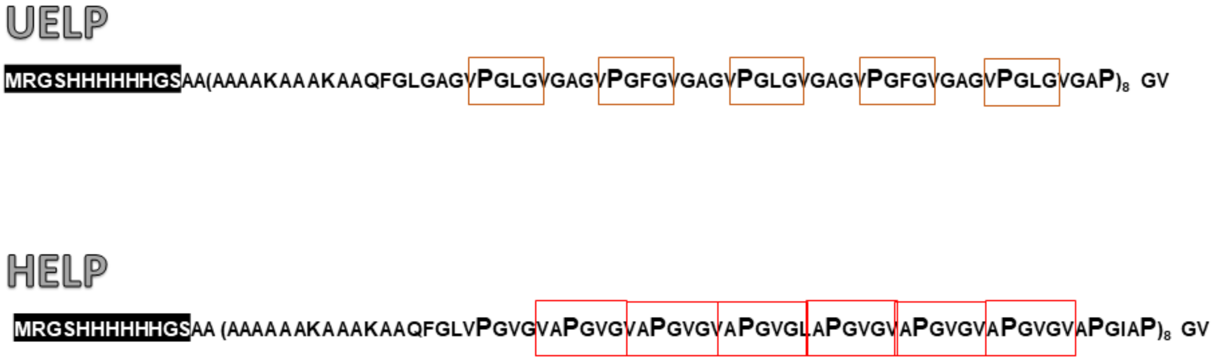

FIGURE 1S

Primary structure of the three human elastin-like polypeptides described in this paper. The monomers (in brackets) are repeated eight times in the final construct. In black, the his-tag at the N-terminus is evidenced. Boxed, the pentapeptidic (orange) and the hexapeptidic (red) repeats.

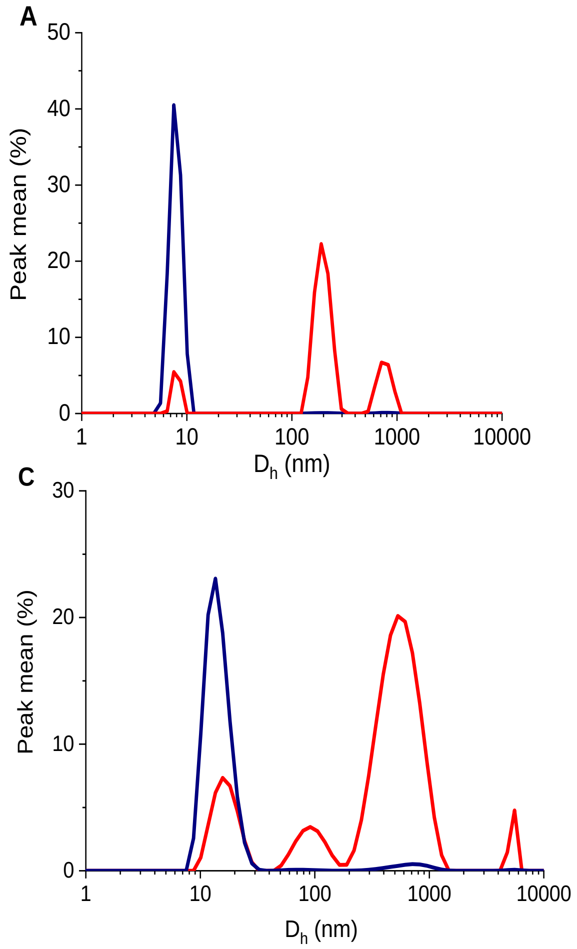

FIGURE 2S

Size distribution of the apparent hydrodynamic diameter (D_h) determined as scattering intensity (red line) and volume (blue line) at 15 °C for the following 2 mg/ml biopolymer solutions: (A) HELP Tris buffer pH 8; (B) HELP Trisl/NaCl 0.15M buffer pH 8; (C) UELP Tris buffer pH8; (D) Tris/NaCl 0.15M pH 8.

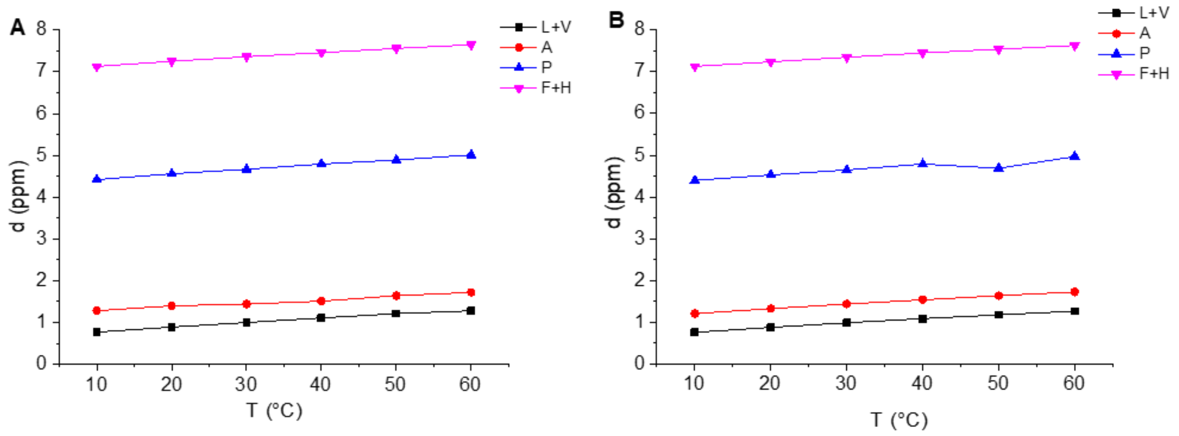

FIGURE 3S

Chemical shift of resonance peaks as a function of temperature for the different amino acidic residues of HELP (A) and UELP (B)

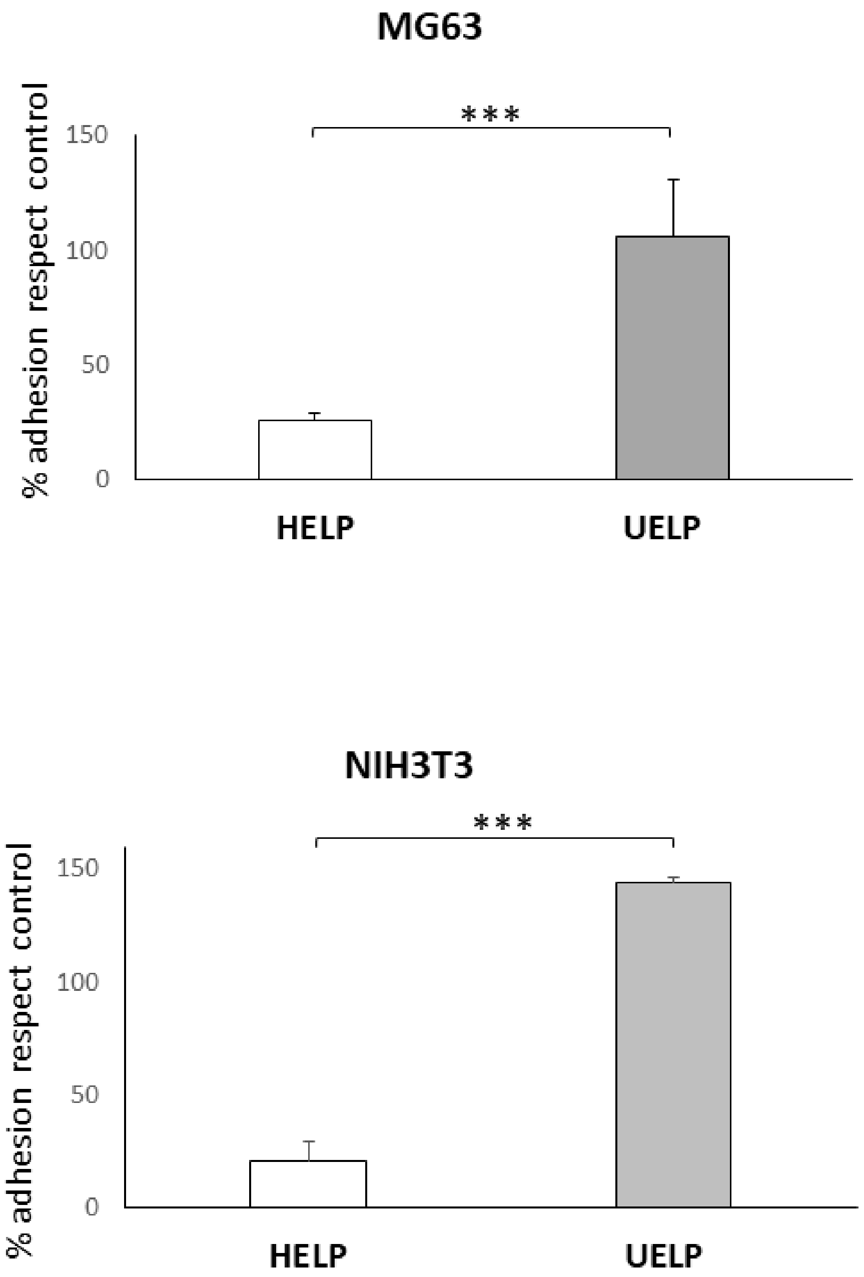

FIGURE 4S

Crystal violet adhesion assays of MG 63 (upper panel) and NIH3T3 (lower panel) cells at 24 hours after seeding. Non-adhesive tissue culture polystyrene wells were left uncoated (control) or were coated HELP and UELP, respectively, like in Figure 10 and Figure 11. The control was the culture on tissue culture treated polystyrene.*** $p < 0.0001$; $n = 5$ via one-way ANOVA.

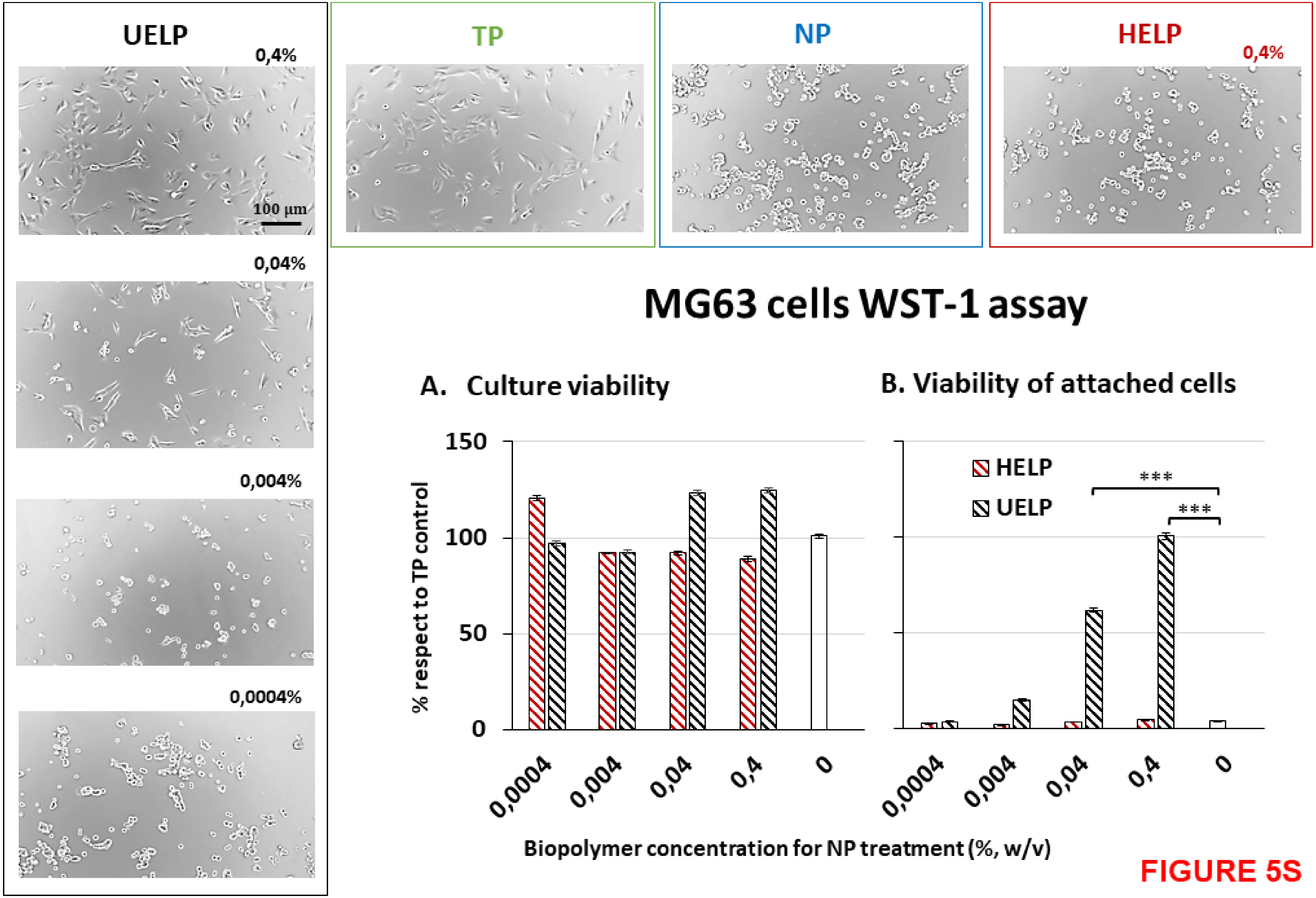
UELP
0,4%
100 µm
0,04%
0,004%
0,0004%
TP
NP
HELP
0,4%
MG63 cells WST-1 assay
A. Culture viability
B. Viability of attached cells
150
100
50
0
% respect to TP control
HELP
UELP
***
***
0,0004
0,004
0,04
0,4
0
Biopolymer concentration for NP treatment (%, w/v)

FIGURE 5S

Representative phase contrast images of MG63 osteoblastic cells of human origin cultured on non treated polystyrene (NP) surfaces coated by incubation with aqueous solutions with different concentration of UELP biopolymer (on the left, boxed in black). Control cultures on tissue culture treated polystyrene (TP, green box), on NP without any biopolymer (blue box) as well as cultures on NP coated with HELP (red box) were carried on in parallel (on the top).

The coatings were prepared in a non-treated 96-well microplate by adding 100 µl of 0.22 µ -filtered aqueous solutions of biopolymer at 0.0004, 0.004, 0.04, and 0.4% (w/v) per well. After overnight incubation at 5°C, the solution was removed, and the wells were washed twice with 200 µL of sterile water and then air-dried. 100 µL of supplemented DMEM containing 5000 cells were seeded in each well. After 24 hours, the cultures were inspected by phase contrast microscopy and the images were acquired.

Cell viability after 24 hours was assessed by the WST-1 metabolic assay. Depending on the coating, after 24 hours two cells morphologies were observed in the cultures: unattached, rounded cells and flat and spread cells, attached to the surface. Thus, the assay was performed under two conditions, one to assess the viability of the culture (attached and unattached cells) and the other to assess the viability of the attached cells only.

A – Culture viability: 24 hours after seeding, 5 µL of WST-1 reagent were directly added to the 100 µL of culture medium in each well. After 90 min of incubation at 37°C, the absorbance was measured at 450 nm by a microplate reader.

B – Viability of the attached cells: 24 hours after seeding, the culture medium was removed and cell were washed with 100 µl of sterile PBS to remove all non-attached cells. Then, 100 µl of supplemented DMEM containing 5 µl of WST-1 reagent were added per well. After 90 min of incubation at 37°C, the absorbance was measured at 450 nm by a microplate reader.

Values were normalized to the TP control cultures.∗∗∗ $p < 0.0001$; $n = 8$ via one-way ANOVA.

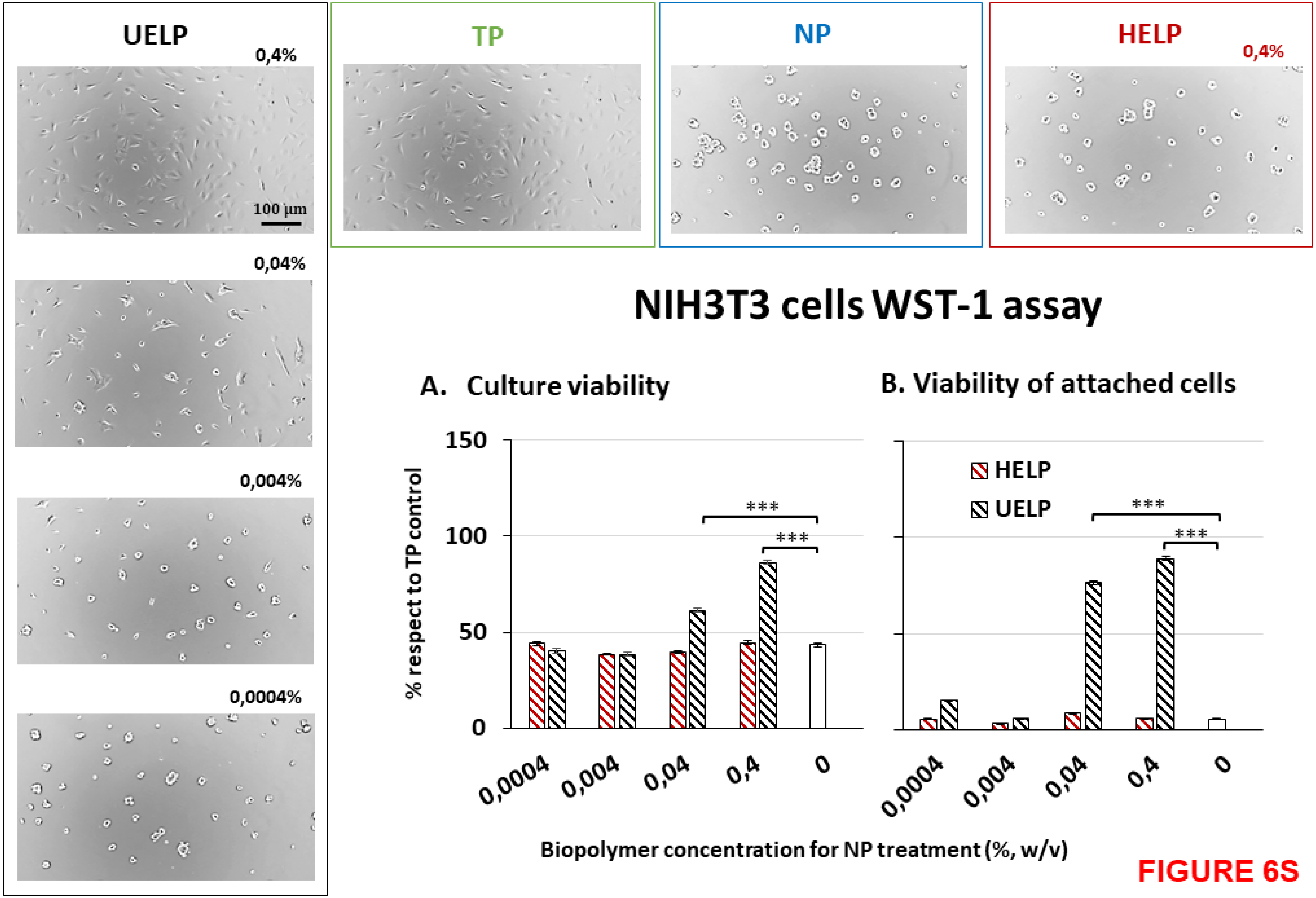
UELP
0,4%
100 µm
0,04%
0,004%
0,0004%
TP
NP
HELP
0,4%
NIH3T3 cells WST-1 assay
A. Culture viability
B. Viability of attached cells
% respect to TP control
150
100
50
0
HELP
UELP
***
***
***
***
0,0004
0,004
0,04
0,4
0
Biopolymer concentration for NP treatment (%, w/v)

FIGURE 6S

Representative phase contrast images of NIH3T3 cells cultured on non treated polystyrene (NP) surfaces coated by incubation with aqueous solutions with different concentration of UELP biopolymer (on the left, boxed in black). Control cultures on tissue culture treated polystyrene (TP, green box), on NP without any biopolymer (blue box) as well as cultures on NP coated with HELP (red box) were carried on in parallel (on the top).

The same conditions and procedures already described for MG63 cell culture in the previous Figure 5S were employed for NIH3T3 murine fibroblasts.

A – Culture viability

B – Viability of the attached cells

Values were normalized to the TP control cultures.*** $p < 0.0001$; $n = 8$ via one-way ANOVA.
